# Demographics and Reported Symptoms Associated With Marijuana Use Among Adolescents and Young Adults

**DOI:** 10.7759/cureus.47844

**Published:** 2023-10-27

**Authors:** Nicole Cumbo, Kaila Lessner, Ciara Marshall, Sayeh Bozorghadad, Susan Boehmer, Robert P Olympia

**Affiliations:** 1 Emergency Medicine, Penn State University College of Medicine, Milton S. Hershey Medical Center, Hershey, USA; 2 Emergency Medicine and Pediatrics, Penn State University College of Medicine, Milton S. Hershey Medical Center, Hershey, USA

**Keywords:** adolescents, cbd, tetrahydrocannabinol (thc), thc, cbd products, marijuana use, marijuana

## Abstract

Purpose

Marijuana use has been increasing in the adolescent population. Our objective was to examine the prevalence of marijuana use among a sample of adolescents and young adults, determine an association with risk-taking behaviors, identify reported medical symptoms, and delineate common beliefs about marijuana use.

Methods

A questionnaire was administered to a sample of patients aged between 12 and 23 years old presenting to the emergency department of Penn State Hershey Medical Center, Hershey, Pennsylvania. Data were stratified by marijuana users and non-users, and further stratified by traditional (vape, pipe, edibles) and non-traditional (oils/concentrates, topical creams) use.

Results

The analysis was based on 200 questionnaires. Thirty-nine percent (n=78) reported marijuana use. Marijuana users were more likely to report previous sexual intercourse (79.5% vs. 32.8%; p=<0.0001), as well as the use of alcohol (50.0% vs. 10.7%; p=<0.0001), cigarettes (41% vs. 8.2%; p=<0.0001), prescription pain medications (20.5% vs. 4.1%; p=0.002), and cocaine (14.1% vs. 0.8%; p=0.0017). Users more likely reported texting while driving (41.0% vs. 13.1%; p=0.005) and experienced physical or electronic victimization due to bullying (43.6% vs. 19.7%; p=0.002). Users were more likely to report gastroesophageal reflux disease (GERD), attention deficit disorder (ADD), anxiety, and depression. The most common symptoms associated with marijuana use were anxiety (65.4%), headache (61.6%), nausea/vomiting (53.8%), cough (51.3%), and abdominal pain (47.4%). Sixty-nine percent of respondents believed marijuana was “safer than other drugs”.

Conclusion

Based on our sample, we identified risk-taking behaviors, medical symptoms, and beliefs associated with marijuana use. Healthcare professionals may use these data to provide screening and anticipatory guidance to adolescents who use marijuana and consider marijuana use in their differential diagnosis.

## Introduction

According to the 2021 Monitoring the Future study, marijuana use by adolescents declined from the late 1990s to the late 2000s but has since begun to rise again, peaking in 2020 with legalization both recreationally and medically in many states [1]. Two major cannabinoids derived from the cannabis plant and sold commercially include tetrahydrocannabinol (THC), the main psychoactive component, and cannabidiol (CBD). The effects felt by the user seem to depend on the THC/CBD content ratio, the mode of intake, and the age of first use [2]. When used recreationally, THC can produce psychoactive symptoms that can range from relaxation and euphoria to depersonalization and decreased memory functions, as well as cognitive and physical symptoms such as hypo- or hypertension as well as appetite changes. The use of THC has also been linked to depression, anxiety, and even suicide among adolescents; however, it is unclear if this is a causal relationship or simply an association [2].

Despite these negative consequences, many benefits of marijuana use have been cited. Clinical indications for medical marijuana vary by state but can include epilepsy and seizure disorders, cancer, HIV/AIDS, neurodegenerative disease, chronic pain, nausea, and post-traumatic stress disorder [3].

In recent years, CBD has quickly grown in popularity in the form of oils, tinctures, and vapes as a treatment for a wide variety of diseases ranging from cancer to epilepsy, anxiety, pain control, and palliative care, with forms of epilepsy being the only FDA-approved indication to date [4]. Many of the risks that must be assessed with CBD oil use deal not necessarily with pure CBD but with unknown contaminants and the overall quality of the CBD oil product. To date, there is no universally accepted method to analyze the quality of CBD products [5]. Side effects are similar to those of THC. Randomized controlled trials using highly purified CBD at therapeutic doses have shown a low abuse potential, although high and supratherapeutic doses had detectable subjective effects [6]. This combination of low cost, low abuse potential, and observed therapeutic effects for epilepsy makes CBD a great option for medical treatment.

While there are many studies documenting different adverse effects associated with marijuana use [7-12], there is little data surrounding the symptoms associated with marijuana use in the adolescent and young adult populations. The purpose of this study is to examine the prevalence of marijuana use among a sample of adolescents and young adults, determine associations with other risk-taking behaviors, and identify clinical symptoms and adverse effects associated with marijuana use. 

## Materials and methods

We conducted an observational, self-administered questionnaire-based study between August 2019 and September 2020, utilizing a sample of 200 patients presenting to the emergency department at the Penn State Hershey Medical Center, Hershey, Pennsylvania, aged 12 and 23 years. We excluded patients presenting with altered mental status, severe pain, developmental delay, acute psychiatric illness, or respiratory distress. Patients were also excluded if they were under 18 years of age, did not have a guardian present, were non-English speakers, or had previously completed the questionnaire.

Once potential subjects were identified, the study team approached the patient and their guardian (for those less than 18 years old), obtained verbal consent, and provided the questionnaire and writing utensil. The questionnaire included 35 questions, focusing on demographics (age, sex, race, sexual orientation, home life, extracurricular activities, social media use), risk-taking behaviors (sexual activity, history of fighting, weapon-carrying, car safety), self-esteem (Rosenberg scale, a validated 10-item scale that measures global self-worth by measuring both positive and negative feelings about the self) [13], medical history, reported medical complaints, frequency of marijuana and other drug use, and perceptions regarding marijuana use (Appendices A-F).

Data were stratified by whether participants were marijuana users or non-users, and marijuana users were further stratified by traditional and non-traditional use. Marijuana users were those who reported marijuana use; non-users were those who did not report marijuana use. Traditional marijuana users were classified as those reporting using vapes, pipes, or edibles, and non-traditional marijuana users were those using oils or concentrates, topical creams, or others.

Descriptive statistics were generated, including means and standard deviations for continuous variables. This was an exploratory survey to find out more information about marijuana, and therefore, no formal sample size calculation was performed. Comparisons between categorical variables were analyzed using contingency table analysis; significance levels were determined by Chi-square tests. Data were stratified by marijuana users and nonusers, and marijuana users were further stratified by traditional and nontraditional use. The internal consistency of Likert scale questions was assessed by Cronbach's alpha, which was found to be 0.89. No adjustments for multiple tests were made. Tests were two-sided and based on a significance criterion of p<0.05. A multivariable logistic regression was then run, including all variables that were significant in the univariate analyses. All analyses were performed with the use of the SAS statistical package, version 9.4 (SAS Inc., Cary, NC). The Pennsylvania State University Institutional Review Board approved this study (approval number: STUDY00012985).

## Results

Data analysis was performed on 200 completed questionnaires. The mean age of respondents was 17.5 years; 62.5% were female, and 66% identified as White (Table 1).

**Table 1:** Demographics of responders, stratified by marijuana users (traditional and non-traditional) and non-users (a) ^a^All data are reported as numerator/denominator, percentage, (95% confidence interval) except "age," which is reported as mean, standard deviation, (95% confidence interval). ^b^Traditional marijuana users were those using vape, pipe, and edibles. ^c^Non-traditional marijuana users were those using oils or concentrates, topical creams, and others.

Demographics		All responses (n=200)	Marijuana users (Traditional and non-traditional) (n= 78)	Marijuana traditional users^b^ (n = 46)	Marijuana non-traditional users^c^ (n=32)	Marijuana non-users (n=122)
Age, in years (mean)		17.5 SD 3.5 (3.2-3.8)	19.4 SD 2.9 (2.4-3.5)	20.1 SD 2.9 (2.4-3.7)	18.5 SD 2.7 (2.1-3.6)	16.3 SD 3.4 (3.0-3.9)
Sex	Female	125/200 (62.5%) (55-69)	46/78 (59.0%) (48-69)	25/46 (54.4%) (40-68)	21/32 (65.6%) (48-80)	79/122 (64.8%) (56-73)
	Male	74/200 (37.0%) (30-44)	32/78 (41.0%) (30-52)	21/46 (45.7%) (32-60)	11/32 (34.4%) (20-52)	42/122 (34.4%) (26-43)
	Other	1/200 (0.5%) (0-3)	0/78 (0.0%) (0-6)	0/46 (0.0%) (0-9)	0/32 (0.0%) (0-13)	1/122 (0.8%) (0-5)
Race	White	132/200 (66.0%) (59-72)	53/78 (68.0%) (57-77)	28/46 (60.9%) (46-74)	25/32 (78.1%) (61-89)	79/122 (64.8%) (56-73)
	Hispanic	31/200 (15.5%) (11-21)	9/78 (11.5%) (6-21)	9/46 (19.6%) (10-33)	0/32 (0.0%) (0-13)	22/122 (18.0%) (12-26)
	African American	17/200 (8.5%) (5-13)	7/78 (9.0%) (4-18)	4/46 (8.7%) (3-21)	3/32 (9.4%) (2-25)	10/122 (8.2%) (4-15)
	Other	10/200 (5.0%) (3-9)	6/78 (7.7%) (3-16)	4/46 (8.7%) (3-21)	2/32 (6.3%) (0-21)	4/122 (3.3%) (1-8)
	Biracial	6/200 (3.0%) (1-7)	2/78 (2.6%) (0-9)	1/46 (2.2%) (0-12)	1/32 (3.1%) (0-17)	4/122 (3.3%) (1-8)
	Asian	4/200 (2.0%) (0-5)	1/78 (1.3%) (0-8)	0/46 (0.0%) (0-9)	1/32 (3.1%) (0-17)	3/122 (2.5%) (0-7)
Sexual orientation	Heterosexual	152/200 (76.0%) (70-81)	62/78 (79.5%) (69-87)	38/46 (82.6%) (69-91)	24/32 (75.0%) (58-87)	90/122 (73.8%) (65-81)
	Bisexual	14/200 (7.0%) (4-11)	7/78 (9.0%) (4-18)	3/46 (6.5%) (1-18)	4/32 (12.5%) (4-29)	7/122 (5.7%) (3-11)
	Not sure	14/200 (7.0%) (4-11)	1/78 (1.3%) (0-8)	0/46 (0.0%) (0-9)	1/32 (3.1%) (0-17)	13/122 (10.7%) (6-18)
	Other	13/200 (6.5%) (4-11)	7/78 (9.0%) (4-18)	4/46 (8.7%) (3-21)	3/32 (9.4%) (2-25)	6/122 (5.0%) (2-10)
	Unanswered	4/200 (2.0%) (0-5)	0/78 (0.0%) (0-1)	0/46 (0.0%) (0-0.1)	0/32 (0.0%) (0-0.2)	4/122 (3.2%) (1-8)
	Homosexual	3/200 (1.5%) (0-5)	1/78 (1.3%) (0-8)	1/46 (2.2%) (0-12)	0/32 (0.0%) (0-13)	2/122 (1.6%) (0-6)
Setting (where responder lives)	Suburban	109/200 (54.5%) (48-61)	43/78 (55.1%) (44-66)	24/46 (52.2%) (38-66)	19/32 (59.4%) (42-74.5)	66/122 (54.1%) (45-63)
	Rural	57/200 (28.5%) (23-35)	23/78 (29.5%) (20-40)	13/46 (28.3%) (17-43)	10/32 (31.3%) (18-49)	34/122 (27.9%) (21-36)
	Inner city	30/200 (15.0%) (11-21)	11/78 (14.1%) (8-24)	8/46 (17.4%) (9-31)	3/32 (9.4%) (2-25)	19/122 (15.6%) (10-23)
	Unanswered	4/200 (2.0%) (0-5)	1/78 (1.3%) (0.01-8)	1/46 (2.2%) (0.01-12)	0/32 (0.0%) (0-13)	3/122 (2.5%) (0.5-7)
Living situation	Two-parent household	90/200 (45.0%) (38-52)	24/78 (30.8%) (22-42)	15/46 (32.6%) (21-47)	9/32 (28.1%) (15-45.5)	66/122 (54.1%) (45-63)
	One-parent household	41/200 (20.5%) (15-27)	15/78 (19.2%) (12-29)	9/46 (19.6%) (10-33)	6/32 (18.8%) (8.5-36)	26/122 (21.3%) (15-29)
	Significant other	25/200 (12.5%) (8.6-18)	15/78 (19.2%) (12-29)	12/46 (26.1%) (15-40)	3/32 (9.4%) (2-25)	10/122 (8.2%) (4-14.6)
	Split	23/200 (11.5%) (8-17)	12/78 (15.4%) (9-25)	4/46 (8.7%) (3-21)	8/32 (25.0%) (13-42)	11/122 (9.0%) (5-15.6)
	Independent	20/200 (10.0%) (6.5-15)	11/78 (14.1%) (8-24)	5/46 (10.9%) (4-23)	6/32 (18.8%) (8.5-36)	9/122 (7.4%) (4-13.6)
	Unanswered	1/200 (0.5%) (0.01-3)	1/78 (1.3%) (0.01-8)	1/46 (2.2%) (0.01-12)	0/32 (0.0%) (0-13)	0/122 (0.0%) (0-4)

Marijuana use was reported in 39% (n=78) of respondents: 59% (n=46) were traditional, and 41% (n=32) were non-traditional. Marijuana users were less likely to live in a two-parent household than non-users (30.8% (95% CI: 22-42) vs. 54.1% (95% CI: 45-63); p= 0.0055).

When stratified by frequency of use, compared to non-users, users were also more likely to have had previous sexual intercourse (79.5% (95% CI: 69-87) vs. 32.8% (95% CI: 25-41.6); p=<0.0001), and have had coitarche before age 18 (53.8% (95% CI: 43-64.5) vs. 21.3% (95% CI: 15-29.5); p=<0.0001) (Table 2).

**Table 2:** Risk-taking behaviors, lifestyle, and other social factors of respondents (a) ^a^All data are reported as numerator/denominator, percentage, (95% confidence interval) except "age," which is reported as mean, standard deviation, (95% confidence interval). ^b^Traditional marijuana users were those using vape, pipe, and edibles. ^c^Non-traditional marijuana users were those using oils or concentrates, topical creams, and others. ^d^Rosenberg self-esteem scale, a validated 10-item scale that measures global self-worth by measuring both positive and negative feelings about the self. EtOH: ethyl alcohol

		All responses (n=200)	Marijuana users (traditional and non-traditional) (n= 78)	Marijuana traditional users^b^ (n = 46)	Marijuana non-traditional users^c^ (n=32)	Marijuana non-users (n=122)
Sexual activity	Has had sexual intercourse previously	102/200 (51.0%) (44-58)	62/78 (79.5%) (69-87)	40/46 (87.0%) (74-94)	22/32 (68.8%) (51-82)	40/122 (32.8%) (25-41)
	Coitarche before 18 years old	68/200 (34.0%) (28-41)	42/78 (53.8%) (43-64)	28/46 (60.9%) (46-73)	14/32 (43.8%) (28-61)	26/122 (21.3%) (15-30)
Lifestyle choices	“Yes” to EtOH use	52/200 (26.0%) (20-32)	39/78 (50.0%) (39-61)	26/46 (56.5%) (42-70)	13/32 (40.6%) (25-58)	13/122 (10.7%) (6-17)
	“Yes” to cigarettes	42/200 (21.0%) (15-27)	32/78 (41.0%) (30-52)	21/46 (45.6%) (32-60)	11/32 (34.4%) (20-52)	10/122 (8.2%) (4-14)
	“Yes” to cigars	28/200 (14.0%) (10-20)	24/78 (30.8%) (22-42)	15/46 (32.6%) (21-47)	9/32 (28.1%) (15-46)	4/122 (3.3%) (1-8)
	“Yes” to chewing tobacco	15/200 (7.5%) (4-12)	14/78 (18.0%) (11-28)	12/46 (26.1%) (15-40)	2/32 (6.2%) (7-21)	1/122 (0.8%) (0-4)
	“Yes” to pain medication abuse	21/200 (10.5%) (7-15)	16/78 (20.5%) (13-31)	12/46 (26.1%) (15-40)	4/32 (12.5%) (4-29)	5/122 (4.1%) (1-10)
	“Yes” to cocaine abuse	12/200 (6.0%) (3-10)	11/78 (14.1%) (8-24)	8/46 (17.4%) (9-31)	3/32 (9.4%) (2-25)	1/122 (0.8%) (0-5)
	“Yes” to inhalant abuse	6/200 (3.0%) (1-7)	3/78 (3.8%) (0-11)	2/46 (4.4%) (0-15)	1/32 (3.1%) (0-17)	3/122 (2.5%) (0-7)
	“Yes” to meth abuse	4/200 (2.0%) (0-5)	3/78 (3.8%) (0-11)	2/46 (4.4%) (0-15)	1/32 (3.1%) (0-17)	1/122 (0.8%) (0-5)
	“Yes” to text and drive	48/200 (24.0%) (18-30)	32/78 (41.0%) (31-52)	25/46 (54.4%) (40-68)	7/32 (21.9%) (11-39)	16/122 (13.1%) (8-20)
	Reports not always wearing a seatbelt	71/200 (35.5%) (29-42)	31/78 (39.8%) (29.6-51)	16/46 (34.8%) (22.6-49)	15/32 (46.9%) (31-63.6)	40/122 (32.8%) (25-41.6)
	“Yes” to physical fighting	40/200 (20.0%) (15-26)	19/78 (24.4%) (16-35)	12/46 (26.1%) (15-40)	7/32 (21.9%) (11-39)	21/122 (17.2%) (11-25)
	“Yes” to weapon carrying	28/200 (14.0%) (10-19)	19/78 (24.4%) (16-35)	12/46 (26.1%) (15-40)	7/32 (21.9%) (11-39)	9/122 (7.4%) (4-13)
Activities	Exercise ( >3 days per week)	130/200 (65.0%) (58-71)	49/78 (62.8%) (52-73)	27/46 (58.7%) (44-72)	22/32 (68.8%) (51-82)	81/122 (66.4%) (57-74)
	Involved in music/art/dance	65/200 (32.5%) (26-39)	32/78 (41.0%) (31-52)	16/46 (34.8%) (22-49)	16/32 (50.0%) (33-66)	33/122 (27.0%) (20-35)
	Involved in team sports	54/200 (27.0%) (21-34)	16/78 (20.5%) (13-31)	10/46 (21.7%) (12-36)	6/32 (18.8%) (8.5-36)	38/122 (31.1%) (23.6-40)
	Involved in extreme sports	26/200 (13.0%) (9-18)	12/78 (15.4%) (9-25)	5/46 (10.9%) (4-23)	7/32 (21.9%) (11-39)	14/122 (11.5%) (7-18)
	Involved in outdoor activities	86/200 (43.0%) (36-50)	44/78 (56.4%) (45-67)	23/46 (50.0%) (36-64)	21/32 (65.6%) (48-80)	42/122 (34.4%) (26-43)
	Social media ( >2 hours per day)	136/200 (68.0%) (61-74)	56/78 (71.8%) (61-80)	36/46 (78.3%) (64-88)	20/32 (62.5%) (45-79)	80/122 (65.8%) (57-73)
	Video games	75/200 (37.5%) (31-44)	33/78 (42.3%) (32-53)	22/46 (47.8%) (34-62)	11/32 (34.4%) (20-52)	42/122 (34.4%) (26-43)
	Television watching (>2 hours per day)	81/200 (40.5%) (34-47)	34/78 (43.6%) (33-54)	26/46 (56.5%) (42-70)	8/32 (25.0%) (13-43)	47/122 (38.5%) (30-47)
	Enjoys spending time with family/friends	167/200 (83.5%) (78-88)	67/78 (85.9%) (76-92)	42/46 (91.3%) (79-97)	25/32 (78.1%) (61-89)	100/122 (82.0%) (74-88)
Self-worth questions^d^	Low self-esteem (score of 15 or below)	50/198 (25.2%) (20-32)	25/77 (32.5%) (23-44)	15/45 (33.3%) (21-48)	10/32 (31.2%) (18-49)	25/121 (20.7%) (19-35)
Frequent co-ingestions (at least once a day)	Coffee	52/200 (26.0%) (20-32)	22/78 (28.2%) (19-39)	12/46 (26.1%) (15-40)	10/32 (31.2%) (18-49)	30/122 (24.6%) (18-33)
	Tea	35/200 (17.5%) (13-23)	13/78 (16.7%) (10-26)	7/46 (15.2%) (7-28)	6/32 (18.8%) (8-36)	22/122 (18.0%) (12-26)
	Soda	42/200 (21.0%) (16-27)	20/78 (25.6%) (17-36)	13/46 (28.3%) (17-43)	7/32 (21.9%) (11-39)	22/122 (18.0%) (12-26)
	Energy drinks	11/200 (5.5%) (3-10)	7/78 (9.0%) (4-18)	5/46 (10.9%) (4-23)	2/32 (6.2%) (0-21)	4/122 (3.3%) (1-8)
Victim of bullying	Neither physically nor electronically	97/200 (48.5%) (42-55)	29/78 (37.2%) (27-48)	16/46 (34.8%) (23-49)	13/32 (40.6%) (25-63)	68/122 (55.7%) (47-64)
	Both physically and electronically	58/200 (29.0%) (23-36)	34/78 (43.6%) (33-55)	20/46 (43.5%) (30-58)	14/32 (43.8%) (28-61)	24/122 (19.7%) (13-28)
	Physically only	24/200 (12.0%) (8-17)	5/78 (6.4%) (2-14)	2/46 (4.4%) (0-15)	3/32 (9.4%) (2-25)	19/122 (15.8%) (10-23)
	Electronically only	20/200 10.0% (6-15)	10/78 (12.8%) (7-22)	8/46 (17.4%) (9-31)	2/32 (6.2%) (0-21)	10/122 (8.2%) (4-14)
	Unanswered	1/200 (0.5%) (0.01-3)	0/200 (0.0%)	0/200 (0.0%)	0/200 (0.0%)	1/122 (0.8%) (0.01-5)

Compared to non-users, users reported significantly more alcohol use (50.0% (95% CI: 39-61) vs. 10.7%% (95% CI: 6-17.5); p=<0.0001), cigarettes use (41% (95% CI: 30-52) vs. 8.2% (95% CI: 4-14); p=<0.0001), cigars use (30.8% (95% CI: 22-42) vs. 3.3% (95% CI: 1-8); p=<0.0001), chewing tobacco use (18% (95% CI: 11-28) vs. 0.8% (95% CI: 0-4); p<0.0001), prescription pain medications without a prescription (20.5% (95% CI: 13-31) vs. 4.1% (95% CI: 1-9); p=0.002), and cocaine use (14.1% (95% CI: 8-24) vs. 0.8% (95% CI: 0.01-5); p=0.0017) (Table 2). Texting while driving was also more likely in marijuana users (41.0% (95% CI: 31-52) vs. 13.1% (95% CI: 8-20); p=0.005), as was carrying a weapon (24.4% (95% CI: 16-35) vs. 7.4% (95% CI: 4-13) p=0.015) (Table 2).

There were no statistically significant differences between users' and non-users’ activities, including exercise, music/art/dance, team sports, extreme sports, social media use greater than two hours per day, outdoor activities, video games, TV watching greater than two hours per day, spending time with family and friends, and low self-esteem (Table 2). There were also no differences observed when investigating frequent co-ingestions such as coffee, tea, soda, and energy drinks. Marijuana users were more likely to be victims of both physical and electronic bullying compared to nonusers (43.6% (95% CI: 33-55) vs. 19.7% (95% CI: 13-28); p=0.002).

Marijuana users were more likely to report the following medical problems: gastroesophageal reflux disease (GERD) (24.4% (95% CI: 16-35) vs. 9.0% (95% CI: 5-15.6); p=0.0164), anxiety (57.7% (95% CI: 47-68) vs. 35.2% (95% CI: 27-44); p=0.007), attention deficit disorder (ADD) (29.5% (95% CI: 20-40) vs. 10.7% (95% CI: 6-17.5); p=0.003), and depression (51.3% (95% CI: 40-62) vs. 27.9% (95% CI: 21-36); p=0.002). There were no significant differences between users and non-users who reported the following symptoms during the previous six months: chest pain, racing heart, difficulty breathing or coughing, dizziness, abdominal discomfort, nausea or vomiting, headache, tremors, sleep disturbances, or dehydration. However, there was a statistically significant increase in reports of anxiety in users as compared to non-users (66% (95% CI: 55-76) vs. 47% (CI: 38-56); p=0.009) (Table 3).

**Table 3:** Medical history and report of symptoms of respondents (a) ^a^All data are reported as numerator/denominator, percentage, (95% confidence interval) except "age," which is reported as mean, standard deviation, (95% confidence interval). ^b^Traditional marijuana users were those using vape, pipe, and edibles. ^c^Non-traditional marijuana users were those using oils or concentrates, topical creams, and others.

		All responses (n=200)	Marijuana users (traditional and non-traditional) (n= 78)	Marijuana traditional users^b^ (n = 46)	Marijuana non-traditional users^c^ (n=32)	Marijuana non-users (n=122)
Medical history	Migraine	64/200 (32.0%) (26-39)	31/78 (39.7%) (29-51)	17/46 )37.0%) (24-51)	14/32 )43.8%) (28-61)	33/122 (27.0%) (20-35)
	Seizures	9/200 (4.5%) (2-8)	4/78 (5.1%) (1-13)	0/46 (0.0%) (0-9)	4/32 (12.5%) (4-29)	5/122 (4.1%) (1-10)
	Heart conditions	14/200 (7.0%) (4-11)	5/78 (6.4%) (2-14)	3/46 (6.5%) (1-18)	2/32 (6.2%) (0-21)	9/122 (7.4%) (4-13)
	Asthma	54/200 (27.0%) (21-33)	17/78 (21.8%) (14-32)	13/46 (28.3%) (17-43)	14/32 (43.8%) (28-61)	37/122 (30.3%) (23-39)
	Cancer	4/200 (2.0%) (0-5)	2/78 (2.6%) (0-9)	0/46 (0.0%) (0-9)	2/32 (6.2%) (0-21)	2/122 (1.6%) (0-6)
	Gastroesophageal reflux disease	30/200 (15.0%) (11-21)	19/78 (24.4%) (16-35)	12/46 (26.1%) (15-40)	7/32 (21.9%) (11-39)	11/122 (9.0%) (5-15)
	Anemia	14/200 (7.0%) (4-11)	8/78 (10.3%) (5-19)	5/46 (10.9%) (4-23)	3/32 (9.4%) (2-25)	6/122 (4.9%) (2-10)
	Thyroid	4/200 (2.0%) (0-5)	2/78 (2.6%) (0-9)	2/46 (4.4%) (0-15)	0/32 (0.0%) (0-13)	2/122 (1.6%) (0-6)
	Diabetes mellitus	4/200 (2.0%) (0-5)	2/78 (2.6%) (0-9)	1/46 (3.1%) (0-12)	1/32 (3.1%) (0-17)	2/122 (1.6%) (0-6)
	Attention deficit disorder	36/200 (18.0%) (13-24)	23/78 (29.5%) (20-40)	13/46 (28.3%) (17-43)	10/32 (31.2%) (18-49)	13/122 (10.7%) (6-17)
	Eating disorder	8/200 (4.0%) (2-8)	6/78 (7.7%) (3-16)	2/46 (4.4%) (0-15)	4/32 (12.5%) (4-29)	2/122 (1.6%) (0-6)
	Anxiety	88/200 (44.0%) (37-51)	45/78 (57.7%) (46-68)	25/46 (54.4%) (40-68)	20/32 (62.5%) (45-77)	43/122 (35.2%) (27-44)
	Depression	74/200 (37.0%) (30-44)	40/78 (51.3%) (40-62)	25/46 (54.4%) (40-68)	15/32 (46.9%) (31-63)	34/122 (27.9%) (21-36)
Reported symptoms in the past six months	Headache	131/200 (65.5%) (59-72)	48/78 (61.6%) (50-71)	28/46 (60.9%) (46-74)	20/32 (62.5%) (45-77)	83/122 (68.0%) (59.3-76)
	Dizziness	92/200 (46.0%) (39-53)	32/78 (41.0%) (31-52)	17/46 (37.0%) (24-51)	15/32 (46.9%) (31-63)	60/122 (49.2%) (40-58)
	Chest pain	62/200 (31.0%) (25-38)	25/78 (32.0%) (23-43)	15/46 (32.6%) (21-47)	10/32 (31.2%) (18-49)	37/122 (30.3%) (23-29)
	Cough	103/200 (51.5%) (44-58)	40/78 (51.3%) (40-62)	25/46 (54.4%) (40-68)	15/32 (46.9%) (31-63)	63/122 (51.6%) (43-60)
	Difficulty breathing	62/200 (31.0%) (25-38)	21/78 (26.9%) (18-38)	12/46 (26.1%) (15-40)	9/32 (28.1%) (15-45)	41/122 (33.6%) (26-42)
	Racing heart	53/200 (26.5%) (21-33)	23/78 (29.5%) (20-40)	13/46 (28.3%) (17-43)	10/32 (31.2%) (18-49)	30/122 (24.6%) (18-33)
	Dehydration	64/200 (32.0%) (26-39)	27/78 (34.6%) (25-46)	16/46 (34.8%) (22-49)	11/32 (34.4%) (20-52)	37/122 (30.3%) (23-29)
	Abdominal discomfort	85/200 42.5% (36-49)	37/78 (47.4%) (37-58)	24/46 (52.2%) (38-66)	13/32 (40.6%) (25-58)	48/122 (39.3%) (31-48)
	Nausea/vomiting	97/200 (48.5%) (42-55)	42/78 (53.8%) (43-64)	25/46 (54.4%) (40-68)	17/32 (53.1%) (36-69)	55/122 (45.1%) (36-54)
	Weakness	82/200 41.0% (34-48)	25/78 (32.0%) (23-43)	14/46 (30.4%) (19-45)	11/32 (34.4%) (20-52)	57/122 (46.7%) (38-55)
	Tremors/shakiness	41/200 (20.5%) (15-27)	19/78 (24.4%) (16-35)	8/46 (17.4%) (9-31)	11/32 (34.4%) (20-52)	22/122 (18.0%) (12-26)
	Sleep disturbances	73/200 (36.5%) (30-43)	32/78 (41.0%) (31-52)	20/46 (43.5%) (30-58)	12/32 (37.5%) (23-55)	41/122 (33.6%) (26-42)
	Anxiety	108/200 (54.0%) (47-61)	51/78 (65.4%) (54-75)	30/46 (65.2%) (51-77)	21/32 (65.6%) (48-80)	57/122 (46.7%) (38-55.5)
Reported symptoms in the past six months that required seeking medical attention	Headache	69/200 (34.5%) (28-41)	28/78 (35.9%) (26-47)	20/46 (43.5%) (30-58)	8/32 (25.0%) (13-42)	41/122 (33.6%) (26-42)
	Dizziness	58/200 (29.0%) (23-36)	20/78 (25.6%) (17-36)	13/46 (28.3%) (17-43)	7/32 (21.9%) (11-39)	38/122 (31.2%) (23-40)
	Chest pain	50/200 (25.0%) (19-31)	22/78 (28.2%) (19-39)	15/46 (32.6%) (21-47)	7/32 (21.9%) (11-39)	28/122 (23.0%) (16-31)
	Cough	61/200 (30.5%) (24-37)	23/78 (29.5%) (20-40)	14/46 (30.4%) (19-45)	9/32 (28.1%) (15-45.5)	38/122 (31.2%) (23-40)
	Difficulty breathing	46/200 (23.0%) (18-29)	17/78 (21.8%) (14-32)	8/46 (17.4%) (9-31)	9/32 (28.1%) (15-45)	29/122 (23.8%) (17-32)
	Racing heart	30/200 (15.0%) (11-21)	10/78 (12.8%) (7-22)	6/46 (13.0%) (6-26)	4/32 (12.5%) (4-29)	20/122 (16.4%) (11-24)
	Abdominal discomfort	65/200 32.5% (26-39)	27/78 (34.6%) (25-46)	15/46 (32.6%) (21-47)	12/32 (37.5%) (23-55)	38/122 (31.2%) (23-40)
	Nausea/vomiting	65/200 32.5% (26-39)	29/78 (37.2%) (27-48)	17/46 (37.0%) (24-51)	12/32 (37.5%) (23-55)	36/122 (29.5%) (22-38)
	Weakness	42/200 21.0% (16-27)	13/78 (16.7%) (10-26)	9/46 (19.6%) (10-33)	4/32 (12.5%) (4-29)	29/122 (23.8%) (17-32)
	Tremors/shakiness	29/200 (14.5%) (10-20)	10/78 (12.8%) (7-22)	6/46 (13.0%) (6-26)	4/32 (12.5%) (4-29)	19/122 (15.6%) (10-23)
	Anxiety	51/200 (25.5%) (20-32)	24/78 (30.8%) (22-42)	17/46 (37.0%) (24-56)	7/32 (21.9%) (11-39)	27/122 (22.1%) (15-30)

The most common symptoms associated with marijuana use were anxiety (65.4%), headache (61.6%), nausea/vomiting (53.8%), cough (51.3%), and abdominal pain (47.4%); there was no significant difference in symptom reports between traditional and nontraditional users.

Vapes (25.6%) and pipes (25.6%) were the most common forms of marijuana use reported, and 61.6% of respondents reported using marijuana recreationally (Table 4).

**Table 4:** Reported use of marijuana products (a) ^a^All data are reported as numerator/denominator, percentage, (95% confidence interval) except "age," which is reported as mean, standard deviation, (95% confidence interval). ^b^Traditional marijuana users were those using vape, pipe, and edibles. ^c^Non-traditional marijuana users were those using oils or concentrates, topical creams, and others.

		Marijuana Users (traditional and non-traditional) (n=78)	Traditional marijuana users^b^ (n=46)	Non-traditional marijuana users^c^ (n=32)
When do they use marijuana?	At night	48/78 (61.5%) (50-71)	32/46 (69.6%) (55-81)	16/32 (50.0%) (33-66)
	Weekends	37/78 (47.4%) (37-58)	24/46 (52.2%) (38-66)	13/32 (40.6%) (25-58)
	Weekdays	27/78 (34.6%) (25-46)	16/46 (34.8%) (22-49)	11/32 (34.4%) (20-52)
	Breaks from school	20/78 (25.6%) (17-36)	14/46 (30.4%) (19-45)	6/32 (18.8%) (8-36)
What form of marijuana do they use?	Vape	20/78 (25.6%) (17-36)	20/46 (43.5%) (30-58)	
	Pipe	20/78 (25.6%) (17-36)	20/46 (43.5%) (30-58)	
	Oils/Concentrates	13/78 (16.7%) (10-26)		13/32 (40.6%) (25-58)
	Other	11/78 (14.1%) (8-24)		11/32 (34.4%) (20-52)
	Topical creams	8/78 (10.3%) (5-19)		8/32 (25.0%) (13-42)
	Edibles	6/78 (7.7%) (3-16)	6/46 (13.0%) (6-26)	
Reason for marijuana use *1 unanswered traditional user	Recreational	48/78 (61.6%) (50-71)	32/46 (69.6%) (55-81)	16/32 (50.0%) (33-66)
	Medical treatment	29/78 (37.2%) (27-48)	13/46 (28.3%) (17-43)	16/32 (50.0%) (33-66)
Where do they get their marijuana?	Store	19/78 (24.4%) (16-35)	9/46 (19.6%) (10-33)	10/32 (31.2%) (18-49)
	Borrowed	9/78 (11.6%) (6-21)	6/46 (13.0%) (6-26)	3/32 (9.4%) (2-25)
	Gave someone else money	7/78 (9.0%) (4-18)	5/46 (10.9%) (4-23.5)	2/32 (6.2%) (0-21)
	Medical card	6/78 (7.7%) (3-16)	4/46 (8.7%) (3-21)	2/32 (6.2%) (0-21)
	Internet	3/78 (3.8%) (1-11)	1/46 (2.2%) (0-15)	2/32 (6.2%) (0-21)
	Took from someone	1/78 (1.3%) (0-7.6)	0/46 (0.0%) (0-9)	1/32 (3.1%) (0-17)
	Other	27/78 (34.6%) (25-46)	18/46 (39.1%) (26-53)	9/32 (28.1%) (15-45)

With respect to perceived beliefs, 64% of respondents believed cannabis products are “less addictive than other drugs,” 69% believed they are “safer than other drugs," 48% believed they “help to treat my medical illness” and 33% believed that marijuana use is “just for fun." When asked to report a myth regarding cannabis products, respondents reported many common phrases, including “it is a gateway drug” (n=11), “it is addictive” (n=9), and “it can kill you” (n=7).

After a multivariable logistic regression of all variables that were significant in the univariate analyses, the variables that ended up being significant in the final model were: bullying, sex, cigars, weakness, ADD, and anxiety (Nagelkerke R-square value for the model was 0.4360, p-value <0.0001, Table 5).

**Table 5:** Odds ratio estimates Nagelkerke R-square value for the model = 0.4360

Variable	Point estimate	95% Wald confidence limit	
Bullying	0.561	0.173 to 1.822	
Sex	0.257	0.119 to 0.553	
Cigars	0.117	0.033 to 0.411	
Weakness	3.073	1.392 to 6.788	
Attention deficit disorder	0.322	0.117 to 0.886	
Anxiety	0.485	0.214 to 1.097	

## Discussion

Based on our sample of adolescents and young adults, we determined that marijuana use was associated with certain risk factors, social factors, victimization of bullying, and certain reported medical history and medical symptoms.

The mean age of users was 19.4 years, and that of non-users was 16.3 years. Cannabis users were less likely to live in a two-parent household than non-users. The mean ages of users and non-users could explain this difference; however, it must also be considered that parental separation or divorce is an adverse childhood experience and can increase the risk of initiation of illicit drug use by two- to four-fold [14].

Cannabis users reported partaking in statistically more risk-taking behaviors, including 10 of the 14 risk-taking behaviors examined: previous sexual intercourse, coitarche before 18 years old, alcohol use, cigarette use, cigar use, chewing tobacco use, prescription medication abuse, cocaine use, texting and driving, and carrying a weapon. Past literature supports our finding: marijuana use in adolescence is associated with risk-taking behaviors [8], including other illicit drug use, risky sexual behavior, bullying, suicidality, and driving while intoxicated. Along with risk-taking behaviors, marijuana use is associated with other drug use disorders going into young adulthood [8, 9]. This supports our conclusion that adolescents using marijuana are endangered by increased participation in risk-taking behavior.

Cannabis users report significantly more physical and electronic bullying victimization than nonusers. One in every five students reports being bullied, which makes the potential consequences of this association even more concerning [15]. Past literature supports this finding: traditional bullying and cyberbullying were significantly associated with marijuana use [10].

Medical histories of GERD, anxiety, ADD, and depression were more likely in marijuana users compared with non-users. Medical marijuana is used for the treatment of GERD, and while research on its efficacy is limited, it does show some potential based on a study by Gottfried et al. [16]. Anxiety was found to be more likely to be reported by non-traditional users compared to traditional users. Current literature says THC relieves anxiety at low doses but increases it at high doses, while CBD decreases anxiety at all doses, so it is possible that the difference in anxiety complaints could be due to the component and dose of cannabis used [11]. Attention deficit disorder was found to be significantly increased in non-traditional users but not in traditional users, and while CBD has been shown to significantly improve hyperactivity in autism spectrum disorder, there is no evidence to support cannabis as a treatment for ADD [17]. Speculation exists due to an association between ADD, the dopamine system, and cannabinoids' influence on dopamine, but chronic use of THC can blunt the dopamine system and worsen ADD [17]. Patients with ADD are more likely to become heavy users and develop cannabis use disorder [17], which could explain why it was found to be increased in our respondents. Lastly, depression was found to be significantly increased in traditional users but not in non-traditional users. Marijuana is known to cause psychotic symptoms, especially in those with a predisposition to mental illness such as schizophrenia, but little is known about the impact of cannabis use on mood and suicidality, much less marijuana as a treatment for depressive symptoms [18].

There were no significant differences in the report of clinical symptoms during the previous six months between users and nonusers, except for anxiety. As discussed, nontraditional users were more likely to have a history of anxiety than traditional users. While THC and CBD are both available in oils, concentrates, and topical creams, CBD oil specifically has been marketed for the relief of anxiety. Non-traditional users have statistically more anxiety, but it is unclear if the anxiety stems from marijuana use or if it is being used to treat pre-existing anxiety in the participants. The increased reports of anxiety among marijuana users show the importance of understanding the efficacy of marijuana in treating various medical conditions and recognizing the adverse effects of cannabis. The use of marijuana and other cannabis products should be added to the differential diagnosis of those presenting with symptoms that may be associated with cannabis use. It is important that marijuana use be considered as an etiology of these symptoms and that patients be appropriately counseled on safe and effective methods to treat their medical conditions.

The perceived beliefs regarding marijuana use could contribute to its growing use among a younger population. Marijuana users and non-users had some similar beliefs and perceived myths about marijuana, highlighting the decrease in disapproval of marijuana seen in the Monitoring the Future Studies [3]. Some of the myths coincide with reality, while others show ignorance of the detrimental effects of marijuana. A myth listed by two respondents was that it can cause memory loss. One of the many effects of marijuana is on memory and the brain [18]. Although many adolescents thought that it was a myth that marijuana is a gateway drug, marijuana use was associated with increased use of almost every drug asked about, and this association is supported by past literature [8, 9]. Many respondents thought that it was a myth that marijuana is addictive (n=9), and although studies have shown CBD to have a low abuse potential when studied in highly purified form, the same cannot be said for cannabis derivatives as a whole [7]. In 2015, the US had 4 million people meet diagnostic criteria for marijuana use disorder, 138,000 voluntarily seek treatment, and studies found that 17% of those who start using in their teens were likely to become dependent on marijuana [19]. Adolescents are growing up during the decriminalization and legalization of both medical and recreational marijuana across the US, which can possibly obscure perceptions of the harmful effects of marijuana by highlighting the positive effects. It is important that physicians meet these perceptions with education and address them appropriately.

As CBD is already legalized, it is growing in popularity as a medical treatment option and is available at local stores. Cannabidiol is marketed for a multitude of medical ailments, and this will only expand as its popularity grows. In the future, THC could be legalized medically and recreationally in more states, so it can be inferred that more adolescents will be using marijuana products for medicinal and recreational purposes. Identifying adolescents using marijuana can allow for patient-physician conversations regarding the safety of use, whether it be for medicinal or recreational purposes.

While our results show the need for more research into marijuana safety and efficacy for medical treatment in the adolescent population, there are important limitations to consider with this particular study. This study was limited by a small sample size due to the global pandemic of 2020 due to SARS-CoV-2 since recruitment was done in the emergency department of a rural trauma center. Since this study was only done at one institution, the findings may not be generalizable. The adolescents’ ability to truthfully complete the questionnaire could have been hindered due to guardians' presence in their emergency department room, and the participants were also patients presenting to the emergency department for medical care, so they could have ongoing medical issues or be more likely to have more medical problems. The potential subjects who declined to participate in the questionnaire were not accounted for, and therefore our conclusions may underestimate reality. Future studies should include other institutions that may represent adolescents and young adults from varying demographics and possibly recruit responders from non-emergency department settings to obtain a more generalizable sense of marijuana use by adolescents and young adults in the US.

Further research on anxiety could be done to understand what motivates this young adult population to use marijuana, especially those using it medicinally. Education regarding the medical uses of CBD oil could be useful in schools to supplement learning about marijuana and other recreational drugs. The biggest drawbacks to its growing use in the medical community are the lack of research and the potential side effects, of which the few we are currently aware include cyclic vomiting syndrome and mental disturbance. Patients and parents should be aware of the risks and benefits of this medical treatment, and more research needs to be done to better articulate the risks of marijuana.

Marijuana use was associated with several risk-taking behaviors, illicit drug use, bully victimization, certain medical problems, and clinical symptoms. Marijuana’s legalization and increasing use among adolescents need to be met with proper counseling to ensure patient safety as different substances and modes of use become readily available. Increasing use of marijuana and other cannabis products should be added to the differential diagnosis of those presenting to the emergency department with symptoms that may be associated with cannabis use. It is important that marijuana use be considered as the etiology of these symptoms.

## Conclusions

This study suggests that marijuana use in adolescents and young adults, regardless of the mode of use, is associated with risk-taking behaviors, bullying victimization, reported past medical history (anxiety, GERD, ADD, and depression), and anxiety complaints. More research is necessary to ensure the safe and appropriate use of marijuana as a plant-derived medication for adolescents and adults alike. Discovering key clinical symptomatology will aid physicians in identifying adolescents using marijuana. Furthermore, physicians should consider marijuana use in adolescents and young adults with high-risk behaviors presenting with anxiety-related complaints or other common symptoms associated with marijuana use, such as headache, nausea/vomiting, cough, and abdominal pain.

## Figures and Tables

**Figure 1:**
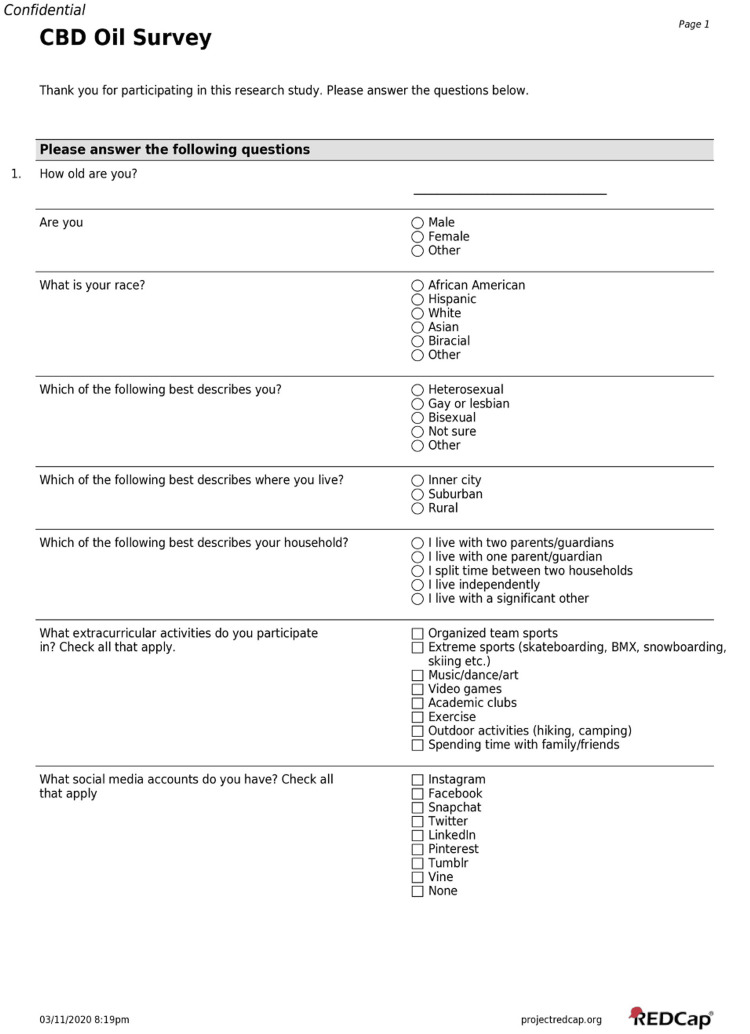
Survey (page 1 of 6)

**Figure 2:**
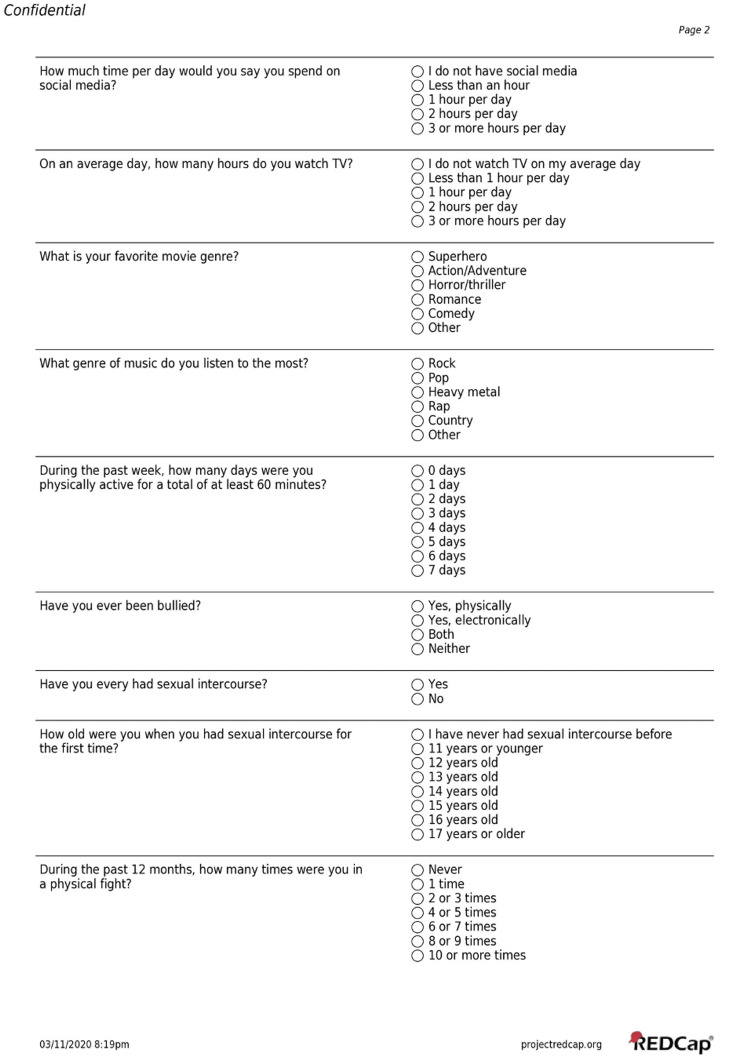
Survey (page 2 of 6)

**Figure 3:**
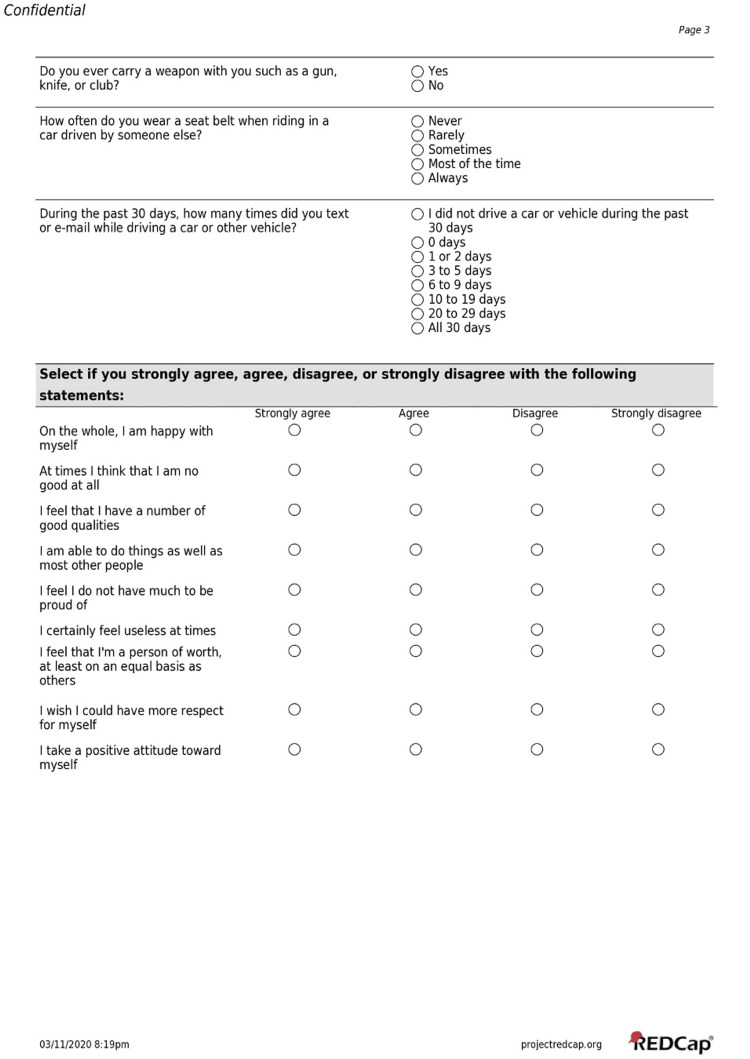
Survey (page 3 of 6)

**Figure 4:**
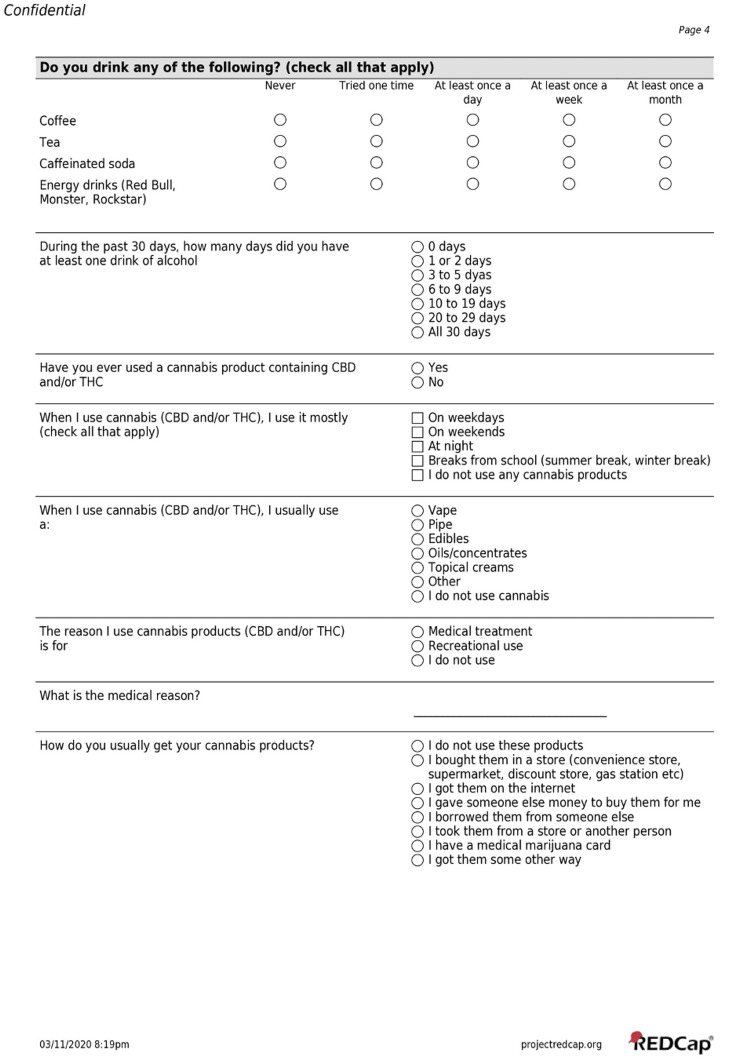
Survey (page 4 of 6)

**Figure 5:**
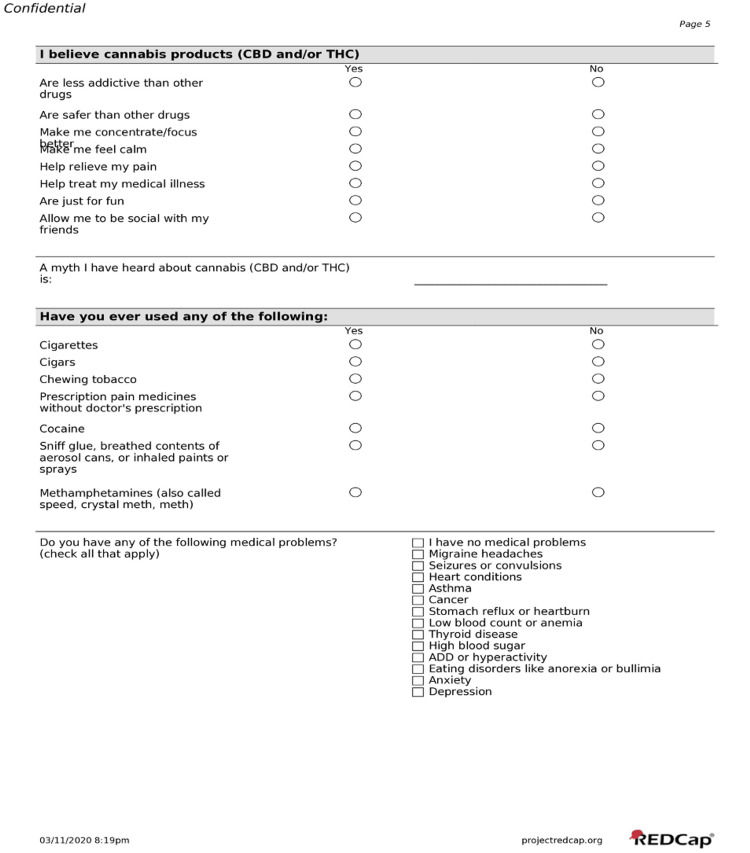
Survey (page 5 of 6)

**Figure 6:**
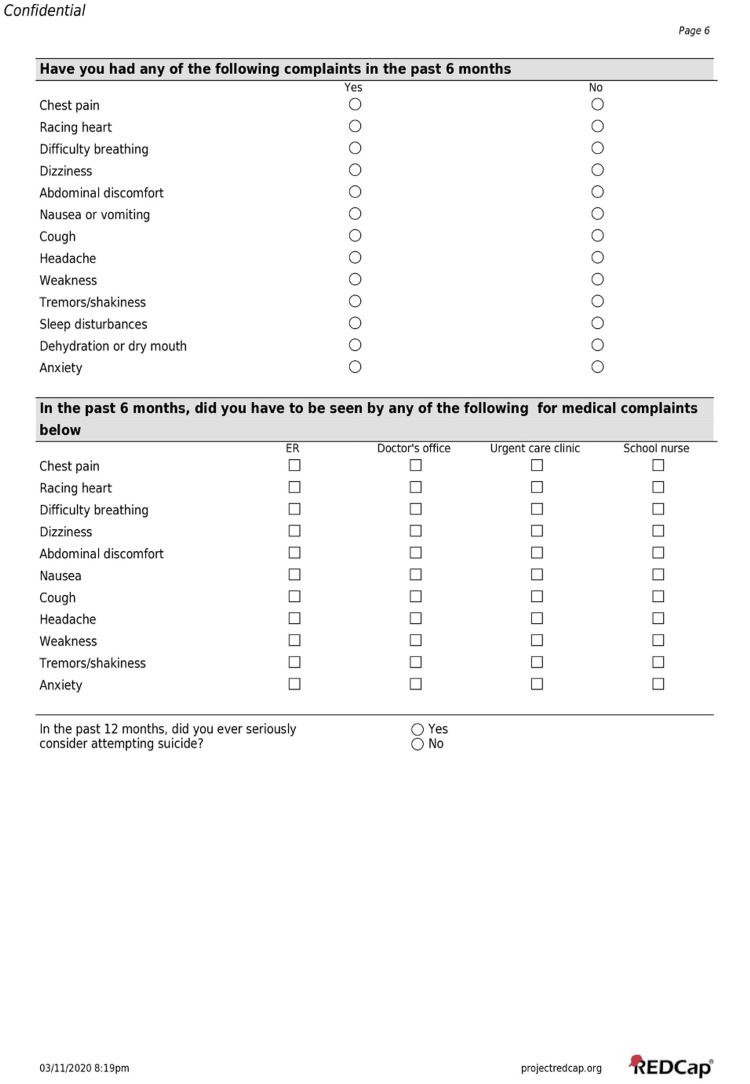
Survey (page 6 of 6)

## Appendices

Appendix A 

Appendix B 

Appendix C 

Appendix D 

Appendix E 

Appendix F 
